# Physiological and Molecular Mechanisms of ABA and CaCl_2_ Regulating Chilling Tolerance of Cucumber Seedlings

**DOI:** 10.3390/plants10122746

**Published:** 2021-12-13

**Authors:** Qian Feng, Sen Yang, Yijia Wang, Lu Lu, Mintao Sun, Chaoxing He, Jun Wang, Yansu Li, Xianchang Yu, Qingyun Li, Yan Yan

**Affiliations:** 1 College of Horticulture, Hebei Agricultural University, Baoding 071000, China; fengqian202112@163.com; 2 Institute of Vegetables and Flowers, Chinese Academy of Agricultural Sciences, Beijing 100081, China; jiaheng1323@163.com (Y.W.); lu580526@icloud.com (L.L.); sunmintao@caas.cn (M.S.); hechaoxing@caas.cn (C.H.); wangjun01@caas.cn (J.W.); liyansu@caas.cn (Y.L.); yuxianchang@caas.cn (X.Y.); 3 College of Horticulture, Henan Agricultural University, 63 Nongye Road, Zhengzhou 450002, China; senyang1989@henau.edu.cn

**Keywords:** ABA, CaCl_2_, cucumber, enzyme activity, low temperature, transcription group

## Abstract

Cold stress is a limiting factor to the growth and development of cucumber in the temperate regions; hence, improving the crop’s tolerance to low temperature is highly pertinent. The regulation of low-temperature tolerance with exogenous ABA and CaCl_2_ was investigated in the cucumber variety Zhongnong 26. Under low-temperature conditions (day/night 12/12 h at 5 °C), seedlings were sprayed with a single application of ABA, CaCl_2_, or a combination of both. Our analysis included a calculated chilling injury index, malondialdehyde (MDA) content, relative electrical conductivity, antioxidant enzyme activities (SOD, CAT, and APX), leaf tissue structure, and expression of cold-related genes by transcriptome sequencing. Compared with the water control treatment, the combined ABA + CaCl_2_ treatment significantly improved the superoxide dismutase (SOD), catalase (CAT), and ascorbate peroxidase (APX) of the seedlings by 34.47%, 59.66%, and 118.80%, respectively (*p* < 0.05), and significantly reduced the chilling injury index, relative electrical conductivity, and MDA content, by 89.47%, 62.17%, and 44.55%, respectively (*p* < 0.05). Transcriptome analysis showed that compared with the water control treatment, 3442 genes were differentially expressed for the combined treatment, 3921 for the ABA treatment, and 1333 for the CaCl_2_ treatment. KEGG enrichment analysis for both the ABA and combined ABA + CaCl_2_ treatments (as compared to the water control) showed that it mainly involves genes of the photosynthesis pathway and metabolic pathways. Differentially expressed genes following the CaCl_2_ treatment were mainly involved in plant hormone signal transduction, plant–pathogen interaction, MAPK signaling pathway–plant, phenylpropanoid biosynthesis, and circadian rhythm–plant. qRT-PCR analysis and RNA-seq results showed a consistent trend in variation of differential gene expression. Overall, this study demonstrated that although all three treatments provided some protection, the combined treatment of ABA (35 mg/L) with CaCl_2_ (500 mg/L) afforded the best results. A combined ABA + CaCl_2_ treatment can effectively alleviate cold-stress damage to cucumber seedlings by inducing physiological changes in photosynthesis and metabolism, and provides a theoretical basis and technical support for the application of exogenous ABA and CaCl_2_ for low-temperature protection of cucumber seedlings.

## 1. Introduction

Cucumber (*Cucumis sativus* L.) is a cold-sensitive plant originating from tropical and subtropical regions [1], that easily suffers damage to growth and development following cold shock [2]. Although China’s cucumber production ranks first in the world, the yield per cultivated area is less than a fifth that of the Netherlands or Israel. As an important economic crop in China, the cultivation area of cucumber in the northern anti-season is increasing [3]. In these situations, long-term cool temperature and short-term critically low temperature in winter and spring lead to physiological and biochemical changes in plants that can result in a substantial loss [4,5,6]. Low-temperature stress can also hinder growth and development in many other vegetable crops [7,8], causing reduced germination rate, general plant damage, and death. In severe cases, cold-induced yellowing and involution on the edges of seedling leaves often causes severe stunting and even death of cucumber plants [9,10]. Long-term low-temperature stress (maximum day temperature less than 20 °C and/or night temperature less than 8 °C) also causes accelerated senescence and decreased yield [11]. More specifically, low-temperature stress leads to changes in biochemical and physiological processes, including the steady state of reactive oxygen species (ROS) [12]. Clearly, any increase in cold tolerance would greatly benefit cucumber production in China.

The phytohormone abscisic acid (ABA), a regulator of plant growth in adverse environments, plays an important role in protection from adverse conditions, including cold stress. ABA synthesis is stimulated under adverse environmental conditions, leading to metabolic changes that increase stress resistance [13,14]. Studies have shown that spraying *Tripterygium wilfordii* seedlings with a 20 mg/L ABA solution can increase the rate of photosynthesis, transpiration, and stomatal conductance of CO_2_, all leading to an improved cold resistance [15]. Similarly, ABA spraying of wheat can increase antioxidant enzyme activity and enhance cold tolerance [14].

After binding to its receptor, Ca^2+^ (a secondary messenger of intracellular function in plants) causes physiological reactions related to enhanced cold resistance. This involves maintenance of membrane integrity (by preventing damage and leakage) and improvement of enzyme activity [16,17,18]. Spraying *Machilus chienkweiensis* with 15 mmol/L of CaCl_2_ causes an increase of soluble sugar, soluble protein, and chlorophyll, thereby improving cold resistance [19]. In Bermudagrass, exogenous application of CaCl_2_ significantly activates both antioxidant enzymes and glutathione to maintain cell ROS at a relatively low level, thereby reducing cell damage caused by cold stress [20].

The above studies demonstrate that leaf application of either ABA or CaCl_2_ can improve resistance to low temperatures, but the use of ABA and CaCl_2_ in combination has not been reported. In this study, cucumber seedlings (variety Zhongnong 26) were grown under low-temperature stress after spraying with different combinations of ABA and CaCl_2_ concentrations. A mixed solution of ABA (35 mg/L) and CaCl_2_ (500 mg/L) significantly enhanced cold resistance and improved growth in cucumber seedlings. Additionally, genes and metabolic pathways related to low-temperature stress response were preliminarily investigated by transcriptome analysis, providing important practical significance.

## 2. Results

### 2.1. Effects of Single and Combined Treatments on Phenoypes of Cucumber Seedlings under Low-Temperature Stress

**Figure 1:**
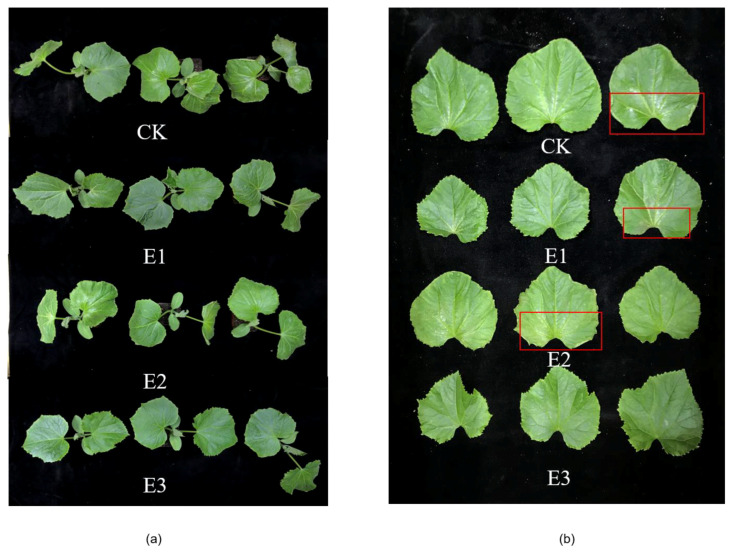
Effects of single and combined treatments on phenotypes of cucumber seedlings under low-temperature stress. (**a**) Overall phenotypic map of cucumber after 72 h cold injury. (**b**) Phenotypic map of all leaves after 72 h cold injury. (CK) Low-temperature stress 72 h, (E1) 25 mg/L ABA, (E2) 1000 mg/L CaCl_2_, and (E3) combined 35 mg/L ABA + 500 mg/L CaCl_2_.

As can be seen in Figure 1, after 72 h of low-temperature stress, the cucumber leaves (the first) appeared dehydrated and wilted after the water control (CK), and the leaf edge was seriously dehydrated and curled upward. Compared with CK, the symptoms of E1 and E2 were alleviated, and the leaves of E3 did not appear to be wilting. The overall comparison of low-temperature stress protection was E3 > E1 > E2 > CK.

### 2.2. Effects of Single and Combined Treatments on Chilling Injury Index and Relative Electrical Conductivity of Leaves under Low-Temperature Stress

**Figure 2:**
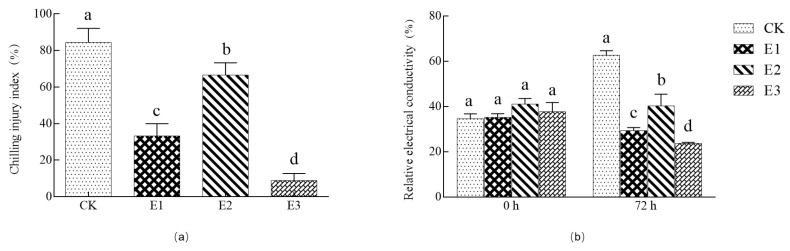
Effects of single and compound treatments on chilling injury index (**a**) and relative electrical conductivity (**b**) of leaves under low-temperature stress. Different small letters indicate significant difference among treatments at the 0.05 level (*n* = 3, *p* < 0.05).

Figure 2a shows the chilling injury index of cucumber seedlings sprayed with 25 mg/L ABA, 1000 mg/L CaCl_2_, and combined 35 mg/L ABA + 500 mg/L CaCl_2_ solutions. Compared to the water control (CK), these seedling treatments had chilling injury indexes that were reduced by 60.53%, 21.05%, and 89.47%, respectively (*p* < 0.05). Similarly, these treatments had relative electrical conductivity measurements significantly lower than the control (53.23%, 35.74%, and 62.17%, respectively) (*p* < 0.05) (Figure 2b). The combined treatment was consistently most effective in reducing both the chilling injury index and the electrolyte leakage, and improved the low-temperature resistance of cucumber seedlings.

### 2.3. Effects of Single and Combined Treatments on Antioxidant Enzyme Activities and MDA Content of Cucumber Seedlings under Low-Temperature Stress

**Figure 3:**
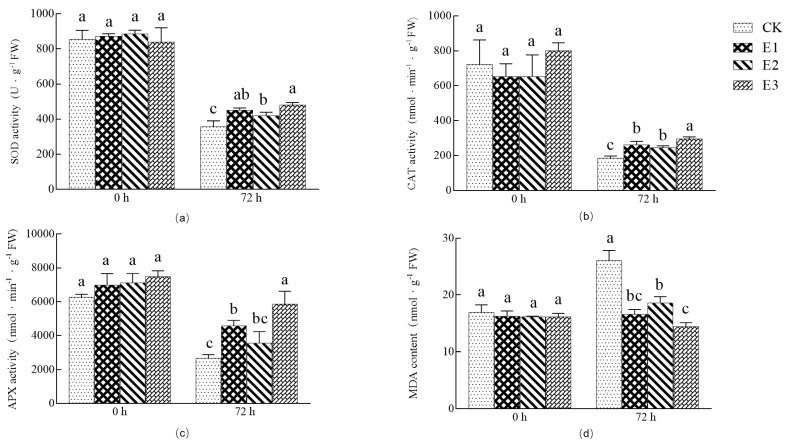
Effects of single and compound treatments on antioxidant enzyme activity (**a**–**c**) and MDA content (**d**) of cucumber seedlings under low-temperature stress. Different small letters indicate significant difference among treatments at the 0.05 level (*n* = 3, *p* < 0.05).

Superoxide dismutase (SOD) and catalase (CAT) scavenge free radicals and can protect cell membranes from toxicity. These enzyme activities directly reflect the adaptability of cells to stress, which is positively correlated with cold resistance. The leaves of cucumber seedlings were sprayed with (E1) 25 mg/L ABA, (E2) 1000 mg/L CaCl_2_, and (E3) combined 35 mg/L ABA + 500 mg/L CaCl_2_ solution (Figure 3a,b). The results show that when the cucumber seedlings were treated with low temperature for 72 h, the SOD enzyme activity of E1, E2, and E3 treatments was significantly increased (26.32%, 17.46%, and 34.47%, respectively) (*p* < 0.05) compared with those of CK. At the same time, compared with CK, the CAT activity of E1, E2, and E3 treatments was significantly increased by 41.20%, 32.15%, and 59.66%, respectively (*p* < 0.05), and the enzyme activity of the E3 treatment was significantly higher than that of other treatments. Ascorbate peroxidase (APX) can effectively reduce the accumulation of reactive oxygen species and reduce cell membrane damage. After 72 h of low-temperature treatment, compared with CK, the APX activities of E1, E2, and E3 treatments increased by 70.93%, 32.80%, and 118.80%, respectively (*p* < 0.05) (Figure 3c). Single treatment and combined treatment can increase the activity of an active oxygen scavenging enzyme, but the compound treatment E3 had the largest effect. At the same time, compared with CK, the malondialdehyde (MDA) content in E1-, E2-, and E3-treated seedlings decreased by 36.30%, 28.71%, and 44.55%, respectively (*p* < 0.05) (Figure 3d), and the E3 treatment was again the best. In summary, E3 treatment increased the antioxidant enzyme activity and APX activity of cucumber seedlings under low-temperature stress, and effectively eliminated the accumulation of MDA content, indicating that E3 treatment had the best effect on improving the low-temperature resistance of cucumber seedlings.

### 2.4. Effects of Single and Combined Treatments on Leaf Anatomical Structure of Cucumber Seedlings under Low-Temperature Stress

**Figure 4:**
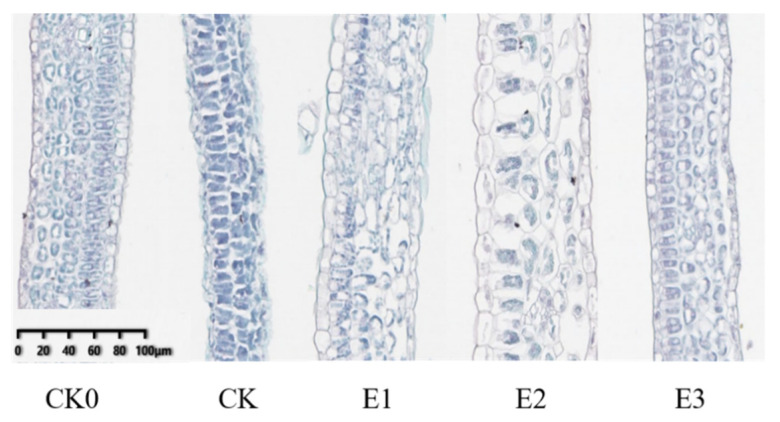
Effects of single and combined treatments on leaf anatomical structure of cucumber seedlings under low-temperature stress. (CK0) Normal temperature, (CK) low-temperature stress 72 h, (E1) 25 mg/L ABA, (E2) 1000 mg/L CaCl_2_, and (E3) combined 35 mg/L ABA + 500 mg/L CaCl_2_. The scale bar is 100 µm.

Figure 4 shows the cross-section of cucumber leaves. The cells of the adaxial palisade tissue treated with CK0 were long and neatly arranged, with a thickness of 16.56~23.25 μm. Compared with CK, the leaf thickness (transverse section) of cucumber seedlings for the three treatments was significantly increased. The E1-treated palisade tissue cells are tightly arranged, and sponge tissue cells are arranged loosely and irregularly, with a thickness of 14.89~21.88 μm. The palisade cells and sponge tissue cells treated with E2 were loosely arranged. The shape of sponge tissue cells were mostly long columnar, irregular, and the intercellular space was large. The thickness was between 22.96 and 28.17 μm. Increased intercellular space speeds up water loss. Compared with E1 and E2, the upper and lower epidermal cells, palisade cells, and spongy tissue cells of E3 treatment were neatly arranged, closely structured, clearly visible, and the overall shape was more regular.

### 2.5. Effects of Single and Combined Treatments on Leaf Tissue Thickness under Low-Temperature Stress

Leaf thickness of single and combined treatments under low-temperature stress is shown in Table 1. The thickness of the lower epidermis, palisade tissue, and leaf thickness of cucumber seedlings treated with E2 were significantly higher than those of CK, E1, and E3 (*p* < 0.05). The upper epidermis and spongy tissue thickness treated with E2 were not significantly different from those of E1 and E3. Comparing E1 and E3 treatments, there was no significant difference in the upper epidermis thickness, lower epidermis thickness, palisade tissue thickness, spongy tissue thickness, and leaf thickness of cucumber seedlings, but they were significantly higher than those of CK. In summary, the effects of E1 and the combined treatment E3 were the best, where the leaf tissue was relatively complete, showing no significant difference from non-temperature stressed seedlings.

### 2.6. Effects of Single and Combined Treatments on the Expression of Antioxidant Enzyme Gene under Low-Temperature Stress

**Figure 5:**
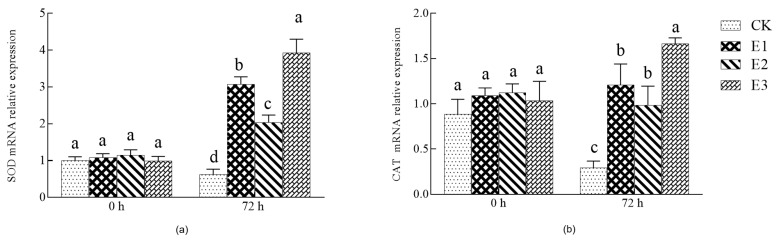
Effects of single and combined treatments on the expression of the antioxidant enzyme gene under low-temperature stress. (**a**) SOD mRNA relative expression. (**b**) CAT mRNA relative expression. Different small letters indicate significant difference among treatments at the 0.05 level (*n* = 3, *p* < 0.05).

As shown in Figure 5, the gene expression of SOD and CAT decreased significantly after 72 h of low-temperature stress (*p* < 0.05). Compared with the water control, E1, E2, and E3 treatments significantly increased the gene expression of SOD and CAT (*p* < 0.05), and the gene expression of SOD and CAT in E3 treatment was significantly higher than that of the E1 and E2 treatments (*p* < 0.05). Again, the E3 treatment had the best effect, indicating that (under cold stress conditions) the combination of ABA and CaCl_2_ can increase the expression of antioxidant enzyme genes, alleviate seedling injury, and improve cold resistance.

### 2.7. Quality Assessment of Sequencing Data

The previous measurements showed that there was no significant difference in each treatment at normal temperature, and that there was a significant difference in each treatment at low temperature. Therefore, we wanted to further explore the response mechanism of each treatment at the transcriptional level at low temperature. Cucumber seedlings with two leaves and one center were sprayed with (CK) water, (E1) ABA, (E2) CaCl_2_, or the compound solution (E3) 35 mg/L ABA + 500 mg/L CaCl_2_. After three days of treatment, the seedlings were subjected to six hours of low-temperature stress at 5 °C before extraction of total RNA. Three biological replicates were made for each treatment, and a total of twelve cDNA libraries were constructed for transcriptome sequencing. Total Reads output for 12 samples averaged 46.22 million, as shown in Table 2. The base number of Q30 in 12 samples was 92.95−94.03% (the sequencing error rate is less than 0.1%), and the GC content ranged from 44.52% to 45.46%. *Cucumis sativus* V2.0 was used as the reference genome for comparison. The comparison rate between each sample and the reference genome was above 95%, indicating that the sequencing data had high quality and could be used for subsequent data analysis.

### 2.8. Analysis of Differentially Expressed Genes

**Figure 6:**
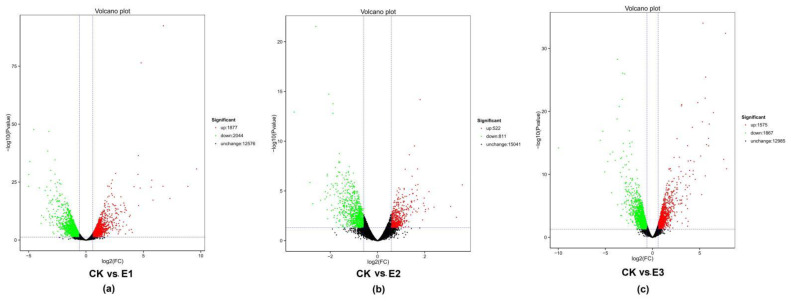
Analysis of DEGs. (**a**) CK vs. E1 volcano plot. (**b**) CK vs. E2 volcano plot. (**c**) CK vs. E3 volcano plot. Notes: Each point in the differential expression volcano plot represents a gene. The abscissa represents the logarithm of the difference in the expression of a certain gene in the two samples, and the ordinate represents the negative logarithm of the statistical significance of the change in gene expression. The larger the absolute value of the abscissa, the greater the multiple difference in the expression amount between the two samples; the larger the ordinate value, the more significant the differential expression, and the more reliable the differentially expressed genes obtained by screening. In the figure, the green dots represent downregulated differentially expressed genes, the red dots represent upregulated differentially expressed genes, and the black dots represent non-differentially expressed genes.

According to the value of FPKM (Fragments per kbp of transcript sequence per mbp sequencing) to reflect the expression of genes, the *p*-value < 0.05 and |log2 Fold Change| > 1.5 were used as screening criteria to obtain differentially expressed genes. The statistical results showed that there were 3921 differential expression genes in CK vs. E1, of which 1877 were upregulated and 2044 were downregulated (Figure 6a). CK vs. E2 had 1333 differentially expressed genes, of which 522 were upregulated and 811 were downregulated (Figure 6b). There were 3442 differential expression genes in CK vs. E3, of which 1575 were upregulated and 1867 were downregulated (Figure 6c). In general, the number of downregulated genes was higher than the upregulated genes for all treatments.

**Figure 7:**
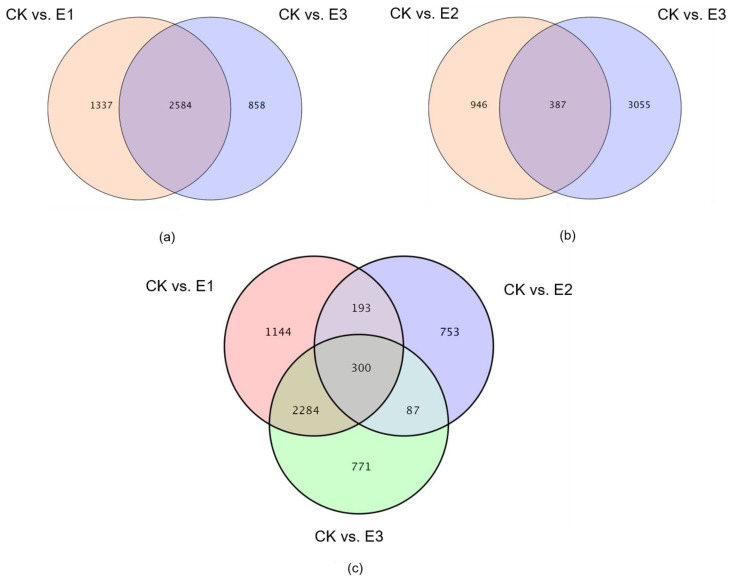
Venn diagram of gene expression. (**a**) CK vs. E1 and CK vs. E3 venn diagram. (**b**) CK vs. E2 and CK vs. E3 venn diagram. (**c**) CK vs. E1, CK vs. E2 and CK vs. E3 venn diagram. Notes: Each circle represents the differential gene in a comparison combination (treatment group vs. control group), the number in the overlapping area of the circle represents the number of common differential genes between the corresponding two or three comparison combinations, and the non-overlapping area represents each comparison combination unique difference gene.

Analyses of differentially expressed genes in E1 and E3 were compared with CK. There were 1337 genes differentially expressed in E1, and 858 genes differentially expressed in E3. There were 2584 differentially expressed genes in E1 and E3, of which 1178 were upregulated and 1406 were downregulated (Figure 7a). Additionally, differentially expressed genes in E2 and E3 compared with CK were analyzed. There were 946 genes differentially expressed in E2, and 3055 in E3. There were 387 differentially expressed genes in E2 and E3, of which 60 were upregulated and 189 were downregulated (Figure 7b). The differentially expressed genes in E1, E2, and E3 compared with CK were analyzed. There were 1144 differentially expressed genes in E1, 753 differentially expressed genes in E2, and 771 differentially expressed genes in E3. There were 300 differentially expressed genes in E1, E2, and E3, of which 55 were upregulated and 143 were downregulated (Figure 7c).

### 2.9. GO Classification Analysis of Differentially Expressed Genes

**Figure 8:**
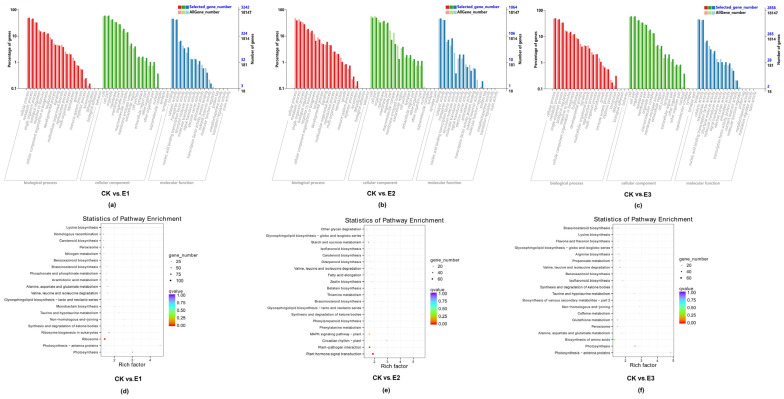
Differentially expressed genes GO classification (**a**–**c**) and enrichment of differentially expressed genes, KEGG (**d**–**f**). Notes: The abscissa is the GO classification, the left of the ordinate is the percentage of the number of genes, and the right is the number of genes (**a**–**c**). Each circle in the figure represents a KEGG pathway, the ordinate indicates the name of the pathway, and the abscissa is the enrichment factor, which indicates the proportion of genes annotated to a pathway among the differential genes and the genes annotated to the pathway among all genes. The larger the enrichment factor, the more significant the enrichment level of differentially expressed genes in this pathway. The color of the circle represents the Q-value, which is the *p*-value after multiple hypothesis testing correction. The smaller the Q-value, the more reliable the significance of the enrichment of differentially expressed genes in the pathway. The size of the circle indicates the number of genes enriched in the pathway: the larger the circle, the more the genes (**d**–**f**).

The DEG obtained by sequencing was subjected to GO (Gene Ontology) functional significance enrichment analysis (Figure 8). The GO process results are divided into three major groups: biological processes, cell components, and molecular functions. For CK vs. E1, a total of 17 items were enriched in the biological process group. In biological processes, cellular processes (1603), metabolic processes (1523), and single-organism processes dominated, followed by biological regulation (614) and response to stimulus (463). A total of 15 items were enriched in the cell component group, and the highly significant expression subclasses were cell (1935) and cell part (1935), followed by organelle (1412) and membrane (1078). A total of 11 items were enriched in the molecular functional groups, and the highly significant expression was binding (1484) and catalytic activity (1390) (Figure 8a). The gene function classifications of CK vs. E1, CK vs. E2, and CK vs. E3 were similar. These accumulated genes indicate that cucumber seedlings undergo complex metabolism and enzymatic reactions during low-temperature stress.

### 2.10. Enrichment Analysis of Differentially Expressed Genes, KEGG

The pathways involved in CK vs. E1, CK vs. E2, and CK vs. E3 differential genes were enriched by KEGG pathway analysis, and DEG was screened with *p*-value < 0.05. In CK vs. E1, the number of DEGs annotated to the ribosome pathway was the largest (142), followed by the photosynthesis pathway (52) and the ribosome biogenesis in eukaryotes (41) (Figure 8d). In CK vs. E2, the number of DEGs annotated to the plant hormone signal transduction pathway was the largest (69), followed by the plant–pathogen interaction (56), the MAPK signaling pathway–plant (38), the phenylpropanoid biosynthesis (26), and the circadian rhythm–plant (17) (Figure 8e). In CK vs. E3, the number of DEGs annotated to the biosynthesis of amino acids pathway was the largest (62), followed by the photosynthesis (43), peroxisome (31), and glutathione metabolism (30) (Figure 8f).

The results of enrichment analysis showed that CK vs. E1 and CK vs. E3 were involved in photosynthesis, metabolic, and other pathways, indicating that the differential genes in photosynthesis-related and metabolic pathways were related to the mechanism of low-temperature stress. CK vs. E2 mainly involves the plant hormone signal transduction pathway, the phenylpropanoid biosynthesis pathway, and the circadian rhythm–plant pathway, indicating that plants can induce cold resistance gene expression and improve cold resistance by regulating the plant hormone signal transduction pathway and by transmitting low-temperature information. The phenylpropanoid biosynthesis is activated under low-temperature stress to promote the accumulation of phenolic compounds which can scavenge harmful reactive oxygen species. The circadian rhythm–plant is closely related to the low-temperature response pathway, forming a feedback regulation loop under low-temperature stress to improve plant frost resistance according to temperature changes.

### 2.11. Quantitative qRT-PCR Verification

**Figure 9:**
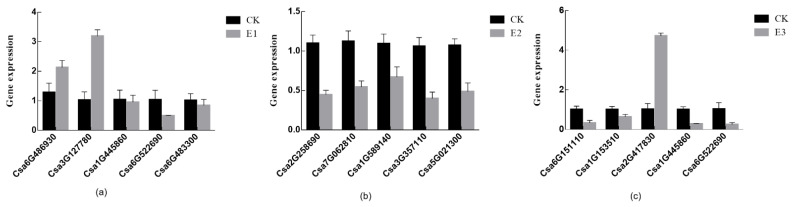
The qRT-PCR validation of differentially expressed genes. (**a**) CK vs. E1-related differential gene expression. (**b**) CK vs. E2-related differential gene expression. (**c**) CK vs. E3-related differential gene expression. (*n* = 3).

To verify the accuracy of the transcriptome sequencing results, five differential genes were randomly selected for qRT-PCR verification in CK vs. E1 (Table 3), CK vs. E2 (Table 4), and CK vs. E3 (Table 5). When the differential gene expression detected by qRT-PCR was compared with the transcriptome sequencing results, the differential gene expression presented by the detection results tended to be consistent, indicating that the sequencing results had high accuracy and reliability (Figure 9).

## 3. Discussion

The cucumber planting scale and yield of China ranks highest in the world, but the yield per cultivated area is only one fifth that of the Netherlands or Israel. In cucumber, chilling and freezing injury can lead to serious yield reduction, or even absolute loss [21]. Studies have shown that even in protected cultivation in winter and spring in China, cucumber plants are still vulnerable to low temperatures, often resulting in poor seed germination, slow seedling growth, and poor plant growth and development [21,22].

Low temperatures can affect various physiological activities of plants, including a weakening of the water absorption capacity. Insufficient internal water can affect normal growth and development, and plants often show symptoms such as leaf curl and wilt that can lead to plant death [23]. Under low-temperature stress, cell membrane permeability can increase and cause metabolic changes and functional disorders. MDA is the main product of cell membrane lipid peroxidation under low-temperature stress. An increase of MDA content can lead to a variety of reactions in plants, damage the membrane structure, and seriously affect its function [24,25]. Under low-temperature stress, the utilization efficiency of O_2_ decreases, and excess O_2_ can be transformed into reactive oxygen species [26]. The accumulation of reactive oxygen species in large quantities can lead to plasma membrane peroxidation, thereby damaging cell membrane structure [27]. Plants must rapidly remove excessive reactive oxygen species through the antioxidant system (namely, SOD, POD, CAT, and APX) [28]. Under low-temperature stress, an increase of antioxidant enzymes is conducive to maintaining low levels of cellular ROS, which can alleviate low-temperature damage to some extent [29]. Low-temperature stress can cause an increase in membrane permeability, electrolyte leakage, leaf relative conductivity, and other physiological disorders that can severely damage seedlings [30].

Studies on the application of ABA and CaCl_2_ alone have been widely reported. ABA is an important hormone relating to abiotic stresses. It can enhance cold resistance by affecting the transcription and post-transcriptional modification of downstream regulatory factors in the signaling pathway [31]. Studies have shown that spraying 0.10 mmol/L of ABA on tomato plants before exposure to 4 °C can promote seedling growth and alleviate leaf wilting [32]. Exogenous ABA can accelerate the synthesis and transportation of natural ABA in plants, regulate plant physiological metabolism, and improve plant resistance to low-temperature stress [33]. Xu et al. [34] have found that exogenous ABA pretreatment can enhance the cold tolerance of pepper seedlings under low temperatures. Zhang et al. [35] have demonstrated that exogenous ABA can reduce the damage of cucumber seedlings caused by low temperature by balancing the production and removal of reactive oxygen species. Wang et al. [36] demonstrated that spraying 20 mg/L of ABA during the flower bud expansion period of apricot can reduce the MDA content and leaf relative conductivity in flowers and young fruits, and effectively prevent the damage caused by spring low temperatures and late frost. The ABA receptor PYR/PYL/RCAR protein, the negative regulator 2C protein phosphatase (PP2C), and the positive regulator SnRK2 are known to be important components of the ABA signal pathway, which together constitute a two-way negative regulatory system for ABA signal transduction and its downstream reactions. The ABA signal induces the PYR/PYL/RCAR protein to interact with PP2C, which leads to the inhibition of PP2C, activates SnRK2, and consequently triggers downstream response genes to be phosphorylated or activated [37]. Overexpression of *OsPYL3* and *OsPYL9* can improve drought and cold stress tolerance of rice [38], and the ABA signal mediated by *PYL-PP2C-SnRK2* was reported to positively regulate banana fruit maturity, cold tolerance, salt tolerance, and osmotic stress [39]. Similarly, CaCl_2_ (a major secondary messenger in plant cells) can regulate plant metabolism and maintain the stability of both the cell membrane and the cell wall. Zhang et al. [40] found that CaCl_2_ alone had a significant effect on improving cold resistance of *Forsythia suspense*. Zhang et al. [41] showed that spraying exogenous Ca^2+^ can reduce the electrolyte permeability and MDA content, increase the soluble sugar content, and reduce the cellular osmotic potential in tomato seedlings, thus improving cold tolerance. The mechanism of exogenous Ca^2+^ improving cold resistance may be similar to that of cold acclimation. During low-temperature acclimation, the calcium ion concentration in the plant cytoplasmic matrix increases and decreases repeatedly, resulting in calcium oscillations [42]. When plants are subjected to low-temperature stress, the concentration of Ca^2+^ in plant cells will increase instantaneously, and the calcium signal encoded by calcium shock can be decoded by calcium binding protein. Calcium binding proteins (CaMs, CBLs-CIPKs, CDPKs, etc.) play an important role in signal transduction, which can transmit the received calcium signals downstream to improve the plant’s resistance to the cold stress [43]. Alcázar et al. [44] found that polyamines and abscisic acid are related to the generation of reactive oxygen species (ROS) signals, which in turn generate nitric oxide that regulates the opening of ion channels and the balance of calcium ions.

Liu et al. [45] studied the effects of salicylic acid (SA), CaCl_2_, and spermidine (Spd) on cold resistance of cucumber seedlings by single and combined treatment. The results showed that the combination of SA, CaCl_2_, and Spd had the best effect, and the three played a synergistic role in improving the cold resistance of cucumber seedlings, but the mechanism was not clear. Zhang et al. [46] combined SA and CaCl_2_ to explore the effect of the mixed solution on cucumber seedlings under complex stress. The results showed that the salt tolerance effect of CaCl_2_ alone was significantly higher than that of SA and CaCl_2_ combined treatment, and the disease resistance effect of SA alone was significantly higher than that of SA and CaCl_2_ combined treatment, so the two showed antagonistic effects. Liu et al. [47] compounded SA and Ca^2+^, and studied the effect of the mixture on the low temperature and weak light tolerance of cucumber. The experimental results showed that the mixture had no synergistic effect or antagonistic effect on the growth tolerance of cucumber seedlings under low temperature and weak light, and the two showed no interference with each other. The results of previous studies on synergistic or antagonistic effects of two or three exogenous substances on plants under stress differ, and the physiological and molecular mechanisms are not clear. However, the effect of ABA and CaCl_2_ compound treatment on the chilling resistance of cucumber seedlings is rarely reported. In this study, ABA and CaCl_2_ were tested individually, as well as in a combined treatment on low-temperature resistance of cucumber seedlings. The results showed that all three treatments effectively reduced the chilling injury index, leaf relative conductivity, and MDA content of cucumber seedlings, and improved the cold tolerance of cucumber seedlings. The combined treatment (E3) had the best effect, and compared with CK, chilling injury index, relative conductivity, and MDA content were significantly reduced by 89.47% (Figure 2a), 62.17% (Figure 2b), and 44.55% (Figure 3d), respectively (*p* < 0.05). In addition, the compound treatment significantly improved the activities of antioxidant enzymes SOD, POD, and APX, indicating that membrane integrity and peroxidation activity were also enhanced.

Low-temperature stress is one of the main environmental pressures that damage metabolic processes and the cell structure of plants [48]. The leaf is the most direct and sensitive tissue to cold exposure, and the storage and regulation of water in leaf mesophyll cells is a favorable means for plants to resist low-temperature stress. With cold stress, the cells of each leaf tissue rapidly regulate and increase water storage, resulting in the increase of leaf cell volume and tissue thickness. In this study, the changes of leaf cell structure of cucumber seedlings under 5 °C treatment were observed by microscopy. The results showed that the leaf thickness of cucumber seedlings decreased significantly after 72 h of low temperature, and that the general leaf structure showed both shrinkage and serious damage. The change of leaf cell structure eventually led to observable leaf curl and wilt. The combined application of ABA and CaCl_2_ protected against this damage, and allowed seedlings to continue with normal growth and development at low temperatures.

The response of plants to any stress is a complex process. The appearance and physiological changes of plants under low-temperature stress are often caused by the internal molecular regulatory network [49]. In this study, high-throughput technology was used to analyze the transcriptome of cucumber seedling leaves stressed at 5 °C with and without application of ABA and CaCl_2_. GO functional annotation of DEGs found that the number of differentially expressed genes increased with the combined treatment, and that the number of upregulated genes was greater than that of downregulated genes, indicating that the compound treatment of exogenous substances can promote the upregulation of functional genes and provide cold resistance of seedlings. KEGG enrichment analysis of DEGs showed that the pathways of CK vs. E1, CK vs. E3, and the control group were significantly enriched. The significant enrichment metabolic pathways of CK vs. E1, CK vs. E3, and the control group were the photosynthesis pathway and the metabolic pathway. The enrichment analysis of the KEGG pathway between CK vs. E2 and the control group showed that the differentially expressed genes were mainly involved in plant hormone signal transduction, plant–pathogen interaction, MAPK signaling pathway–plant, phenylpropanoid biosynthesis, and circadian rhythm–plant.

Based on the physiological indexes and transcriptome analysis, the compound effect of exogenous substances in this study was better. We found that the best combination of mixed liquid is not necessarily the optimum concentration of a single substance. We used the chilling injury index, MDA content, relative conductivity, SOD, and CAT activities to screen out that the optimal concentration for ABA treatment alone was 25 mg/L (Supplementary Figure S1). This is consistent with the findings of Duan et al. [32]. The optimum concentration of CaCl_2_ was 1000 mg/L (Supplementary Figure S2), which was consistent with the results of Liu et al. [45]. However, the optimum concentration of combined treatment was 35 mg/L ABA + 500 mg/L CaCl_2_ (Supplementary Figure S3). In order to further verify that the combined optimum treatment (E3) was superior to the ABA single optimum treatment (E1) and CaCl_2_ single optimum treatment (E2), we measured the chilling injury index, MDA content, relative conductivity, SOD, CAT, and APX activities, respectively, and found that the effect of E3 treatment was superior to that of E1 and E2 treatments, but there was no significant difference in MDA content and SOD activity between E3 treatment and E1 treatment. Compared with E1 and E2 treatments, the upper and lower epidermal cells, palisade cells, and spongy tissue cells of E3 treatment were neatly arranged, closely structured, and clearly visible, and the overall shape was more regular. Finally, we conducted transcriptome analysis on CK, E1, E2, and E3 treatments, and found that a large number of common differential genes were screened out in E1 and E3 treatments, indicating that there were some identical regulatory pathways between them, and they were enriched together to photosynthesis, metabolism, and other pathways. These results suggest that the differential gene expressions in photosynthesis-related and metabolic pathways are related to the mechanism of low-temperature stress. Exogenous ABA plays a major role in it, indicating that exogenous ABA can regulate low-temperature signaling pathways in plants and improve cold tolerance of plants. In this study, the response mechanism of cucumber seedlings to low-temperature stress was comprehensively analyzed at the physiological and transcriptional levels, providing a theoretical basis of low-temperature tolerance regulation pathways, as well as cold acclimation of cucumber.

## 4. Materials and Methods

### 4.1. Test Materials and Growth Conditions

Cucumber seeds (variety Zhongnong 26) were purchased from Zhongshu Seed Industry Technology Co., Ltd. (Beijing, China) ABA and CaCl_2_ were purchased from Beijing Huayueyang Biotechnology Co., Ltd. (Beijing, China). Environmentally controlled growth testing was conducted in environmental chambers of the Institute of Vegetables and Flowers, Chinese Academy of Agricultural Sciences, from May 2020 to May 2021.

### 4.2. Experimental Design

Seeds were briefly soaked in water for 30 min at 55 °C, followed by 4 h at room temperature, and then left to germinate for 24 h at 28 °C. Seeds with uniform germination were selected and planted in peat and vermiculite (2:1 by volume) for growth with conditions as follows: 24 °C/12 h daytime, 18 °C/12 h night, light intensity 500 μmol·m^−2^·s^−1^, and 60–80% relative humidity. When the seedlings grew to two leaves and one heart, seedlings with uniform growth were transplanted to larger containers (7 × 7 × 8 cm) for experimental treatments. For each treatment, 3 mL of solution was sprayed evenly on the leaves. Water was sprayed on control plants (designated CK). All spraying occurred at 9:00 am for three consecutive days. Plants were then transferred to a sunlit greenhouse for one day, and then to controlled conditions for low-temperature stress treatment. Here, the photoperiod was 12 h of light and 12 h of dark at a constant temperature of (5 ± 0.5 °C) [50,51,52]. At 72 h, the chilling injury index, malondialdehyde (MDA) content, relative electrical conductivity, and antioxidant enzyme activities under different treatments were measured.

#### 4.2.1. Single Treatment Concentration Screening of ABA and CaCl_2_

Test solutions were either ABA or CaCl_2_ at specific concentrations. ABA concentrations were 15, 25, and 35 mg/L (designated S1, S2, and S3, respectively). CaCl_2_ concentrations were 500, 1000, and 1500 mg/L (designated S4, S5, and S6, respectively). Water (CK) was sprayed as a control. At 72 h, the chilling injury index, MDA content, relative electrical conductivity, SOD, and CAT activities were measured (Supplementary Figures S1 and S2). The results showed that the best concentration of ABA was 25 mg/L (Supplementary Figure S1), and the best concentration of CaCl_2_ was 1000 mg/L (Supplementary Figure S2).

#### 4.2.2. Combined Treatment Concentration Screening of ABA and CaCl_2_

Nine mixed solutions of both ABA and CaCl_2_ are listed in Table 6. Water (CK) was sprayed as a control. At 72 h, the chilling injury index, MDA content, relative electrical conductivity, SOD, and CAT activities were measured (Supplementary Figure S3). The results showed that the optimum concentration of combined treatment was 35 mg/L ABA + 500 mg/L CaCl_2_ (Supplementary Figure S3).

#### 4.2.3. Effects of Single and Combined Treatments on Chilling Resistance

The preliminary experiment showed that the optimal concentration of ABA alone was 25 mg/L, that of CaCl_2_ alone was 1000 mg/L, and that of the combined treatment was 35 mg/L ABA + 500 mg/L CaCl_2_. The experimental solutions were tested: (E1) ABA alone at 25 mg/L, (E2) CaCl_2_ alone at 1000 mg/L, and (E3) the compound solution of 35 mg/L ABA + 500 mg/L CaCl_2_. Again, the control spray was water (CK). In addition to the measurements recorded for the above experiments, at 72 h, the activity of APX was measured, and the anatomical structure of the first functional leaves under cucumber growth point was observed by paraffin section. At low-temperature stress for 6 h, RNA was extracted from the first functional leaves below the growth point of cucumber for transcriptome analysis.

### 4.3. Determination Indexes and Methods

#### 4.3.1. Determination of Chilling Injury Index

Referring to the methods of Yu et al. [53], chilling injury symptoms of seedlings can be graded as follows: Level 0 was harmless. Level 1 showed leaves slightly shriveled, margin yellowed, or slightly dehydrated in lower first or second leaves, while 3rd and new leaves were unharmed. Level 2 showed leaf shrinkage. The leaf margin of the first and second leaves was seriously dehydrated. The leaf margin of the third leaf was yellow or slightly dehydrated. There was no obvious damage to the heart leaf. Level 3 showed dehydration spots in the middle of the first and second leaves, severe dehydration at the edge of the third leaf, and slight dehydration at the heart leaf. Level 4 showed dehydration spots in the middle of the first and second leaves, wilted leaves, dehydration spots in the middle of the third leaves, and water loss in the heart leaves. However, the heart leaves could still recover at room temperature. Level 5 included severe dehydration and wilting of all leaves. Furthermore, the seedlings could not recover at room temperature.

Chilling Injury Index = (1 × R1 + 2 × R2 + 3 × R3 + 4 × R4 + 5 × R5 + 0 × R0)/(Number of plants per treatment × 5)

R0~R5 are the number of seedlings of grade 0~5, respectively.

#### 4.3.2. Determination of Relative Electrical Conductivity

The relative electrical conductivity was assayed based on the method described by Jiang and Zhang [54]. The relative electrical conductivity (*REC*) was calculated using the following formula:

*REC* (%) = (*EC*1/*EC*2) × 100% (1)

where *EC*1 and *EC*2 refer to the initial electrical conductivity and final electrical conductivity, respectively.

#### 4.3.3. Determination of MDA Content and Antioxidant Enzyme Activity

The content of malondialdehyde (MDA) was determined by the thiobarbituric acid (TBA) colorimetric method, the activity of superoxide dismutase (SOD) was determined by the nitroblue tetrazolium (NBT) method, the activity of catalase (CAT) was determined by UV spectrophotometer, and the content of ascorbic acid peroxidase (APX) was determined by the kit provided by Suzhou Keming Biotechnology Co., Ltd., Suzhou, China.

#### 4.3.4. Observation of Paraffin Sections of Cucumber Leaves

The sampling position was the first functional leaf under the cucumber growing point.

We used the paraffin section method as described by Lu [55], by cutting the right of the leaf (0.5 × 1 cm) to ensure repeated samplings were taken from the same position. The leaf was placed in a vial containing approximately 4 mL of 70% standard fixative (FAA) and was vacuumed and stored in a refrigerator at 4 °C for subsequent use. The leaves were cut into sections and subjected to dehydration, transparency, wax immersion, embedding, sectioning, safranine, and green double staining. The leaf thickness, upper epidermis, lower epidermis, fence tissue, and sponge tissue were then scanned and measured. We used an OLYMPUS microscope to measure the data, with an average of nine fields of view.

#### 4.3.5. RNA-Seq Method

After total RNA extraction, the purity, concentration, and integrity of RNA samples were measured and digested, before transcriptome sequencing analysis using an Illumina HiSeq2500 high-throughput sequencing platform. The gene expression measurement index was determined by the FPKM method, and the differential expression between samples was analyzed by edgeR. *p*-value < 0.05 and |log2 Fold Change| > 1.5 were used as the screening criteria to obtain differentially expressed genes. Finally, GO functional annotation and KEGG enrichment analysis of differentially expressed genes were performed.

#### 4.3.6. Real-Time Quantitative PCR

Total RNA was extracted from cucumber leaves using Trizol reagent (Beijing Huayue Biotechnology Co., Ltd., Beijing, China.) and cDNA was synthesized using M-MuLV reverse transcriptase (Beijing Huayue Biotechnology Co., Ltd., Beijing, China.). Primers of cucumber differentially expressed genes related to low-temperature stress were designed by NCBI software and synthesized by Tsingke Biotechnology Co., Ltd., Beijing, China. The relative quantitative analysis of RNA genes in the samples was performed using the 7500 RT-PCR System (ABI, Applied Biosystems, Carlsbad, CA, USA). Actin was used as the internal reference gene, and the expression level of the target gene was analyzed using the 2^−ΔΔCt^ method. Three repeats per gene were performed. The primers for gene determination are shown in Table 7.

### 4.4. Data Processing

Microsoft Excel 2010 and GraphPad Prism 6 software were used for data processing and mapping. SPSS 17.0 software was used for one-way analysis of variance, and the Duncan test was used for multiple comparisons of significant differences (*p* < 0.05).

## Figures and Tables

**Table 1:** Effects of single and combined treatments on leaf tissue thickness under low-temperature stress.

Treatments	Upper Epidermal	Lower Epidermal	Palisade Tissue Cells	Spongy Tissue Cells	Thickness of Leaf
CK0	9.54 ± 1.30 a	7.11 ± 0.62 a	19.81 ± 2.16 b	44.35 ± 6.15 a	78.20 ± 2.10 b
CK	6.64 ± 1.57 b	6.36 ± 0.54 a	13.76 ± 0.88 c	27.88 ± 6.90 b	55.18 ± 11.43 c
E1	8.68 ± 1.85 ab	6.87 ± 1.42 a	17.62 ± 2.31 b	43.08 ± 5.78 a	77.77 ± 10.48 b
E2	10.53 ± 1.34 a	8.56 ± 0.31 b	25.07 ± 1.93 a	48.23 ± 0.80 a	96.21 ± 1.64 a
E3	8.83 ± 0.73 ab	6.85 ± 0.35 a	19.52 ± 1.54 b	41.46 ± 0.10 a	75.95 ± 3.20 b

Different small letters indicate significant difference among treatments at the 0.05 level (*n* = 9, *p* < 0.05).

**Table 2:** Transcriptome sequencing quality.

Samples	Total Reads	Clean Reads	Clean Bases	GC Content	% ≥ Q30	Mapped Reads	Uniq Mapped Reads	Multiple Map Reads	Reads Map to ‘+’	Reads Map to ‘−’
CK-1	44,618,310	22,309,155	6,670,561,854	45.07%	92.95%	42,447,730 (95.14%)	41,467,828 (92.94%)	979,902 (2.20%)	21,163,186 (47.43%)	21,160,656 (47.43%)
CK-2	49,222,556	24,611,278	7,342,704,106	45.46%	93.19%	47,198,149 (95.89%)	46,176,017 (93.81%)	1,022,132 (2.08%)	23,486,116 (47.71%)	23,516,941 (47.78%)
CK-3	47,991,182	23,995,591	7,161,992,462	45.40%	93.39%	46,006,438 (95.86%)	44,979,931 (93.73%)	1,026,507 (2.14%)	22,862,658 (47.64%)	22,925,469 (47.77%)
E1-1	46,593,340	23,296,670	6,962,313,210	44.52%	93.63%	44,751,389 (96.05%)	43,819,837 (94.05%)	931,552 (2.00%)	22,278,382 (47.81%)	22,300,619 (47.86%)
E1-2	41,426,060	20,713,030	6,191,201,142	44.54%	93.54%	39,811,450 (96.10%)	38,945,058 (94.01%)	866,392 (2.09%)	19,785,979 (47.76%)	19,820,662 (47.85%)
E1-3	47,878,380	23,939,190	7,158,833,810	44.56%	93.57%	45,923,173 (95.92%)	44,898,067 (93.78%)	1,025,106 (2.14%)	22,858,624 (47.74%)	22,869,652 (47.77%)
E2-1	61,136,178	30,568,089	9,125,858,838	45.17%	93.70%	58,691,249 (96.00%)	57,195,375 (93.55%)	1,495,874 (2.45%)	29,188,498 (47.74%)	29,248,521 (47.84%)
E2-2	39,594,192	19,797,096	5,917,625,040	45.23%	93.61%	38,070,280 (96.15%)	37,202,631 (93.96%)	867,649 (2.19%)	18,922,921 (47.79%)	18,960,436 (47.89%)
E2-3	38,751,786	19,375,893	5,792,658,928	45.03%	93.40%	36,989,221 (95.45%)	36,190,762 (93.39%)	798,459 (2.06%)	18,380,305 (47.43%)	18,422,299 (47.54%)
E3-1	46,552,240	23,276,120	6,950,477,954	44.87%	93.09%	44,571,839 (95.75%)	43,602,148 (93.66%)	969,691 (2.08%)	22,165,634 (47.61%)	22,194,830 (47.68%)
E3-2	46,518,224	23,259,112	6,948,491,590	44.74%	94.03%	44,610,658 (95.90%)	43,662,215 (93.86%)	948,443 (2.04%)	22,228,239 (47.78%)	22,246,386 (47.82%)
E3-3	39,071,642	19,535,821	5,825,747,050	44.53%	93.47%	37,427,027 (95.79%)	36,655,676 (93.82%)	771,351 (1.97%)	18,634,330 (47.69%)	18,656,942 (47.75%)

Note: (1) Samples: Sample analysis number; (2) Total Reads: The number of Clean Reads, calculated as single-ended; (3) Clean reads: The total number of pair-end Reads in Clean Data; (4) Clean bases: The total number of bases in Clean Data; (5) GC content: Clean Data GC content, that is, the percentage of G and C bases in the Clean Data to the total bases; (6) ≥Q30%: The percentage of bases whose Clean Data quality value is greater than or equal to 30; (7) Mapped Reads: The number of Reads compared to the reference genome and the percentage in Clean Reads; (8) Uniq Mapped Reads: The number of Reads compared to the unique position of the reference genome and the percentage in Clean Reads; (9) Multiple Map Reads: The number of Reads compared to multiple locations in the reference genome and the percentage in Clean Reads; (10) Reads Map to ‘+’: The number of Reads compared to the positive strand of the reference genome and the percentage in Clean Reads; (11) Reads Map to ‘−’: The number of Reads aligned to the negative strand of the reference genome and the percentage in Clean Reads.

**Table 3:** CK vs. E1-related differential genes.

Gene ID	*p*-Value	log_2_ Fold Change	Regulation	Forward Primer Sequence (5′–3′)	Reverse Primer Sequence (5′–3′)
*Csa6G486930*	2.50 × 10^−12^	1.58103343	up	GGCTCAGTCTCTGGTTGCTT	GGTCACACTCACTGGTCACA
*Csa3G127780*	1.39 × 10^−6^	1.715534501	up	AGGAGGTTCATCCATGCCAA	GGCATTGTCATCAGTGGGTG
*Csa1G445860*	3.30 × 10^−22^	−3.093382086	down	TTCCCAGAGCTTCTTTCCCG	GCTAGAATGCTTTGGGCGTG
*Csa6G522690*	2.64 × 10^−15^	−2.402005435	down	AGTTGAAGAATGGAAGGTTGGC	GCCAAGTTTTCCAAAGGACCC
*Csa6G483300*	6.61 × 10^−13^	−1.52695047	down	TCTCCTGGGGTTGTGGGTTA	GCAGTCCTCCTTCCATCGTT
*Actin*	/	/	/	TTCTGGTGATGGTGTGAGTC	GGCAGTGGTGGTGAACATG

**Table 4:** CK vs. E2-related differential genes.

Gene ID	*p*-Value	log_2_ Fold Change	Regulation	Forward Primer Sequence (5′–3′)	Reverse Primer Sequence (5′–3′)
*Csa2G258690*	0.013669262	−1.744223754	down	AGCTGCCAAGCAAGTTCTCA	TTCTGTCGGTTTCTCCCACG
*Csa7G062810*	0.010998375	−1.612041763	down	AGCAACCATCTGTCTGGCTC	TCTCTTTTGGCAGCACACCA
*Csa1G589140*	0.008039942	−1.594646598	down	ATGGAAGGCAACACCCAGTT	CATTCTCCTGCTCTTTAACACCT
*Csa3G357110*	8.40 × 10^−5^	−2.407987186	down	AACCCCCAAAGTCCGATGAC	ACTCCGTGTGGTACTCCTCA
*Csa5G021300*	1.36 × 10^−7^	−1.602384465	down	GAGTGGCGCAGAGTAAGTGT	ACTGCCACATCCCCCAAAAA
*Actin*	/	/	/	TTCTGGTGATGGTGTGAGTC	GGCAGTGGTGGTGAACATG

**Table 5:** CK vs. E3-related differential genes.

Gene ID	*p*-Value	log_2_ Fold Change	Regulation	Forward Primer Sequence (5′–3′)	Reverse Primer Sequence (5′–3′)
*Csa6G151110*	2.27 × 10^−6^	−1.584198668	down	CTTTTCATTCCGTCGCCGTC	TAACCGACGCAACAACTCCA
*Csa1G153510*	0.002037282	−1.570926378	down	CTCGGGGATGATAAGCAGCA	ACAGAGCCAATGCCGATCTT
*Csa2G417830*	1.27 × 10^−6^	2.11518284	up	GGGGTTGCCAGGTTCATACA	TAGTTGCGATGGATGCTCCC
*Csa1G445860*	2.14 × 10^−6^	−2.81315738	down	TTCTAGCCATTTGGGCCTCC	TGGGTAGATGGGATCGGTCA
*Csa6G522690*	0.000125907	−2.135788485	down	ATCTGGGCTTGCCAGGTTG	TGCCAACCTTCCATTCTTCAACT
*Actin*	/	/	/	TTCTGGTGATGGTGTGAGTC	GGCAGTGGTGGTGAACATG

**Table 6:** ABA and CaCl_2_ compound combined treatment and concentration.

Treatments	Concentration
CK	Fresh water
T1	15 mg/L ABA + 500 mg/L CaCl_2_
T2	25 mg/L ABA + 500 mg/L CaCl_2_
T3	35 mg/L ABA + 500 mg/L CaCl_2_
T4	15 mg/L ABA + 1000 mg/L CaCl_2_
T5	25 mg/L ABA + 1000 mg/L CaCl_2_
T6	35 mg/L ABA + 1000 mg/L CaCl_2_
T7	15 mg/L ABA + 1500 mg/L CaCl_2_
T8	25 mg/L ABA + 1500 mg/L CaCl_2_
T9	35 mg/L ABA + 1500 mg/L CaCl_2_

**Table 7:** Real-time PCR primer sequence.

Gene	Forward Primer Sequence (5′–3′)	Reverse Primer Sequence (5′–3′)
*ZnSOD*	ACGGGTAATGTTTCTGGTCTCAA	GGAATCTGGCAGTCAGTAATGGT
*CAT*	ACCCACCTTACTTGTGCTGATTT	TCATACCATCACGGACGAAAAAT
*Actin*	TTCTGGTGATGGTGTGAGTC	GGCAGTGGTGGTGAACATG

## Data Availability

Data are contained within the article or supplementary material.

## Supplementary Materials

The following are available online at https://www.mdpi.com/article/10.3390/plants10122746/s1, Figure S1: Effects of ABA alone on chilling injury index (A), relative electrical conductivity (B), antioxidant enzyme activity (C,D) and MDA content (E) of cucumber seedlings under low temperature stress. Figure S2: Effects of CaCl_2_ alone on chilling injury index (A), relative conductivity (B), antioxidant enzyme activity (C,D) and MDA content (E) of cucumber seedlings under low temperature stress. Figure S3: Effects of combined treatment on chilling injury index (A), relative conductivity (B), antioxidant enzyme activity (C,D) and MDA content (E) of cucumber seedlings under low temperature stress.
