# Supplementary figures and images for: Physiological and Molecular Mechanisms of ABA and CaCl2 Regulating Chilling Tolerance of Cucumber Seedlings
Source: Plants (Basel). 2021 Dec 13;10(12):2746. doi: 10.3390/plants10122746 (PMC8705041; doi:10.3390/plants10122746)

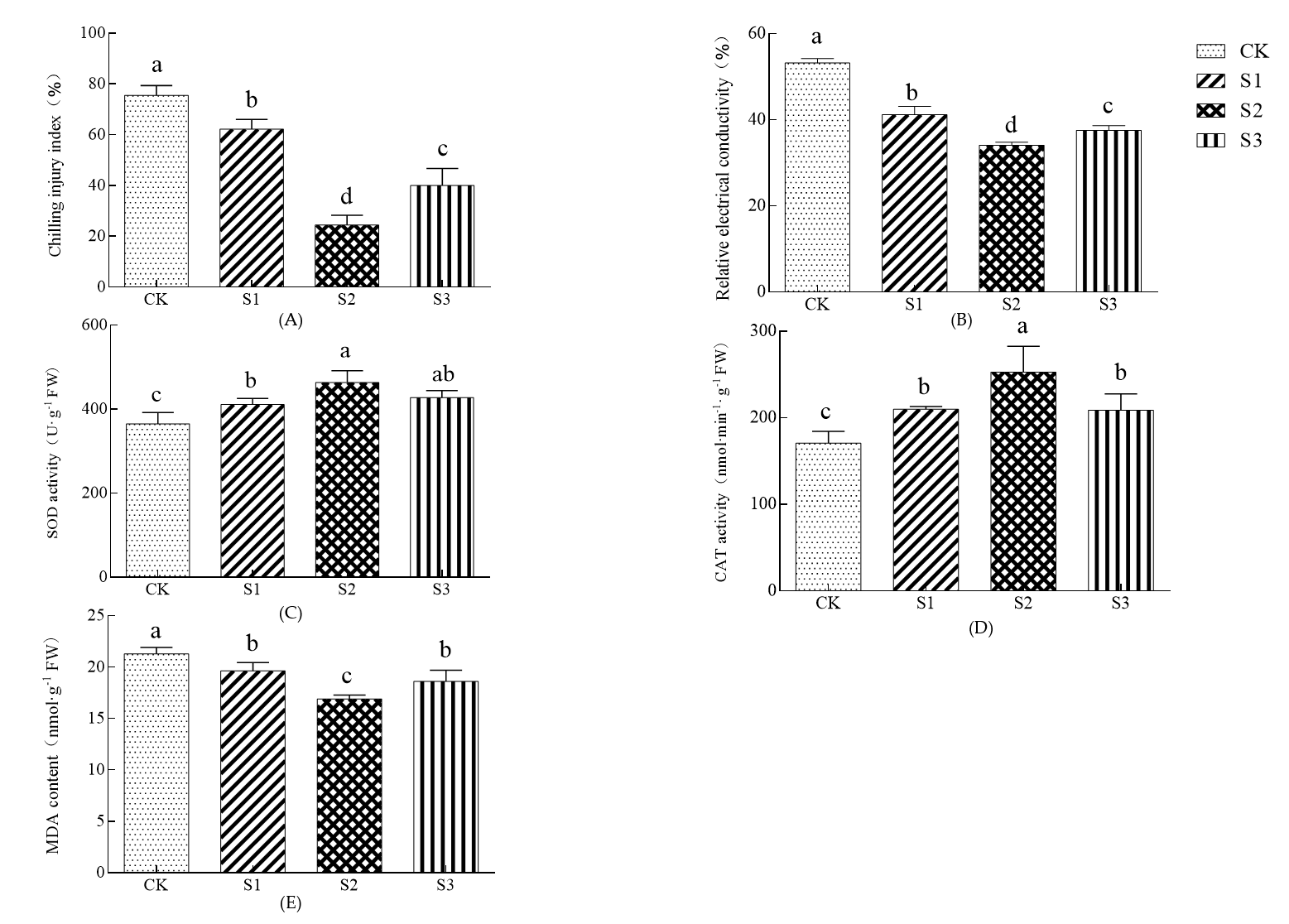

Supplement: Supplementary file 1 [file plants-10-02746-s001.zip › plants-1476493-SI/Figure S1.png]

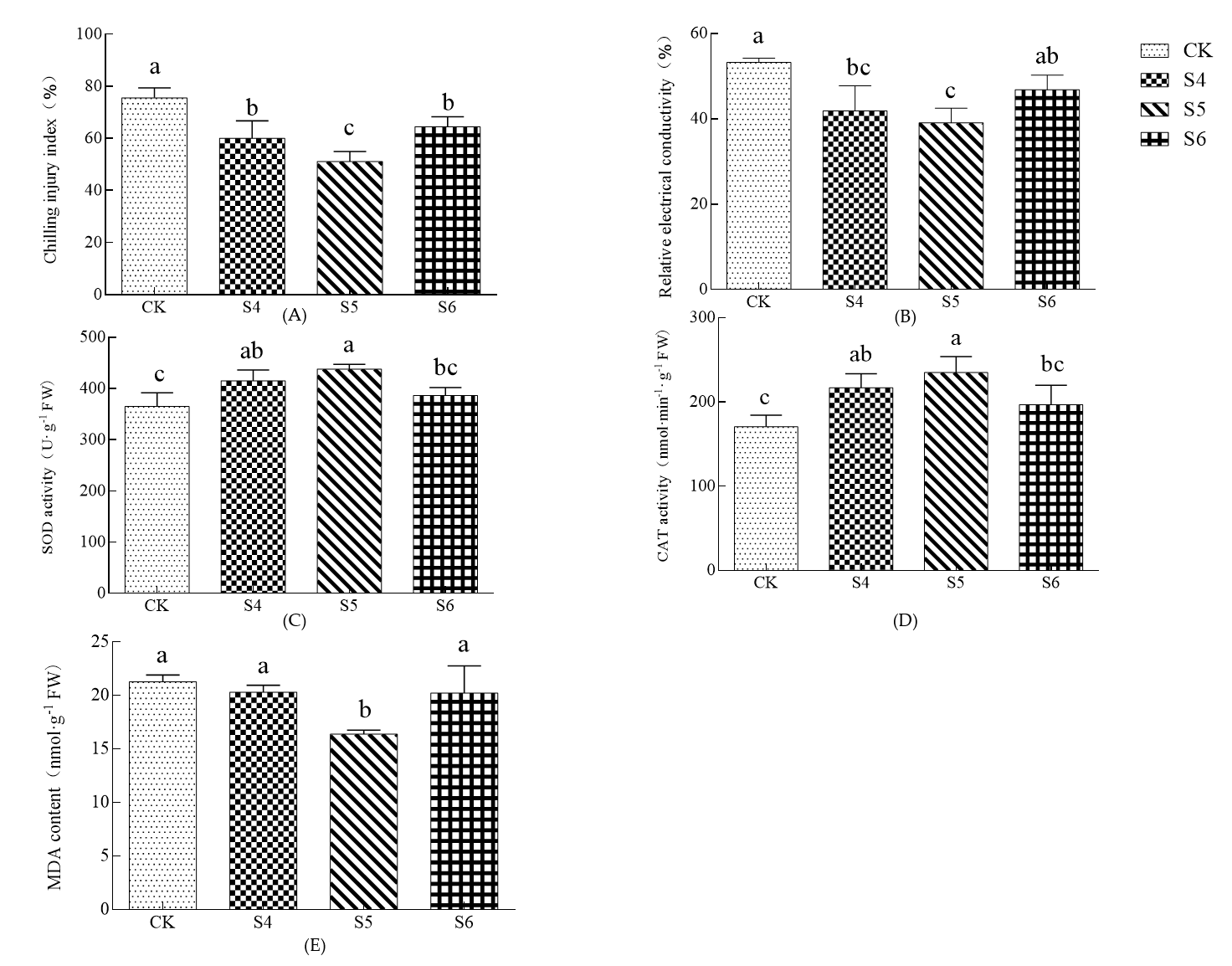

Supplement: Supplementary file 1 [file plants-10-02746-s001.zip › plants-1476493-SI/Figure S2.png]

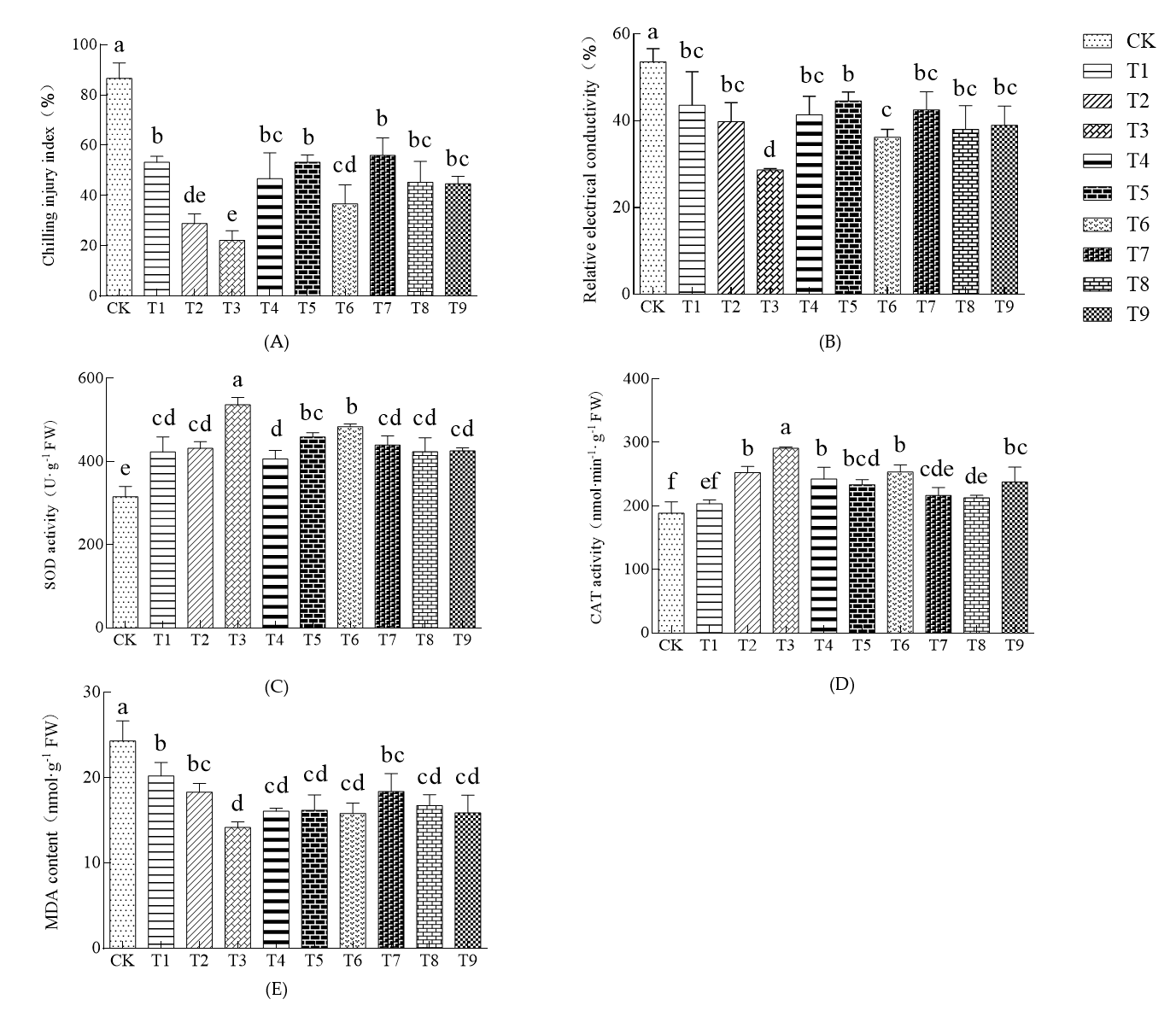

Supplement: Supplementary file 1 [file plants-10-02746-s001.zip › plants-1476493-SI/Figure S3.png]
